# Assessing the impact of mega-city construction engineering on urban livability: an explorative study of Yan'an

**DOI:** 10.3389/fpubh.2024.1358872

**Published:** 2024-05-15

**Authors:** Lei Liu, Lanyue Zhang, Yimeng Guo, Keang Ren

**Affiliations:** ^1^Neijiang Normal University, Neijiang, Sichuan, China; ^2^Chengdu University of Technology, Chengdu, Sichuan, China; ^3^Sichuan University Jinjiang College, Meishan, Sichuan, China; ^4^Sichuan Institute of Administration, Chengdu, Sichuan, China

**Keywords:** urban livability, Mountain Excavation and City Construction (MECC), factor analysis, multiple linear regression, determining factor, squeezed city

## Abstract

Yan'an City is a typical squeezed city in China and faces the challenge of limited living space. The adoption of the “Mountain Excavation and City Construction (MECC)” program was poised to elevate the city's livability. Despite the importance of megacity construction projects, few studies have examined their impact on urban livability. This study aims to fill this gap by analyzing the effects of MECC and the satisfaction characteristics of urban livability in Yan'an City, based on survey data from both old and new urban areas. Employing factor analysis and multiple linear regression, this paper assesses the influence of MECC on urban livability across different demographic groups, including age, educational background, and occupation. The empirical findings demonstrate a significant positive effect of the MECC project on urban livability. However, during categorization discussions, some respondents expressed concerns about its negative impact. The results of multiple linear regression indicate that factors such as career prospects, residential satisfaction, interpersonal relationships, and transportation level significantly influence livability (R2 = 0.607 in ND and R^2^ = 0.609 in OD).

## 1 Introduction

The World Health Organization put forward the four fundamental concepts of safety, health, convenience, and comfort of the living environment in 1961, which provided a valuable reference for evaluating livable cities (1). Previous studies have found that the intimate link between the quality of urban environments and personal wellbeing, with consequential impacts on a city's competitiveness and developmental trajectory (2–4). Adverse urban conditions, exemplified by air pollution leading to respiratory ailments (5) and extreme temperatures posing risks of heat stress (6), detrimentally affect residents' quality of life. Consequently, fostering livable urban environments has emerged as a paramount objective within China's new urbanization agenda. The Chinese government has proactively charted novel approaches to realize modern, livable cities that cater to the diverse material and cultural needs of its populace. Thus, a nuanced understanding of factors contributing to regional livability satisfaction is pivotal for informing urban development strategies and augmenting residents' overall life satisfaction.

With the acceleration of urbanization, major engineering construction plays a crucial role in the development of cities. These projects have been shown to affect various aspects of livability significantly. Transportation infrastructure improvements have reduced congestion and travel time (7). Developing green spaces as part of these projects contributes to urban residents' overall wellbeing and health, promoting a more sustainable and livable urban environment (8). Additionally, major infrastructure projects have the potential to attract investment and stimulate economic growth, creating job opportunities and enhancing the overall conditions of cities (9, 10). However, major engineering construction also brings many environmental problems and challenges, including land use, resource consumption, and soil erosion (11–13).

As rivers and valleys surround the city, the widest zone of Yan 'an is < 1 km, and the narrowest region is only more than 0.2 kilometer wide. With sustained population growth, the population density of some areas even exceeds that of Beijing and Shanghai in this city. The urban layout puts some buildings next to the mountains in danger of landslides. Moreover, with the increasing density of buildings, negative impacts such as congestion, air pollution, noise, and medical resources shortage also gradually appear in some areas. These problems lead to reduced urban livability and hinder regional sustainable development (14, 15). In this context, local governments have introduced targeted measures to promote urban construction actively. MECC engineering has become essential to solving existing problems and promoting innovative urban development.

**Figure 1:**
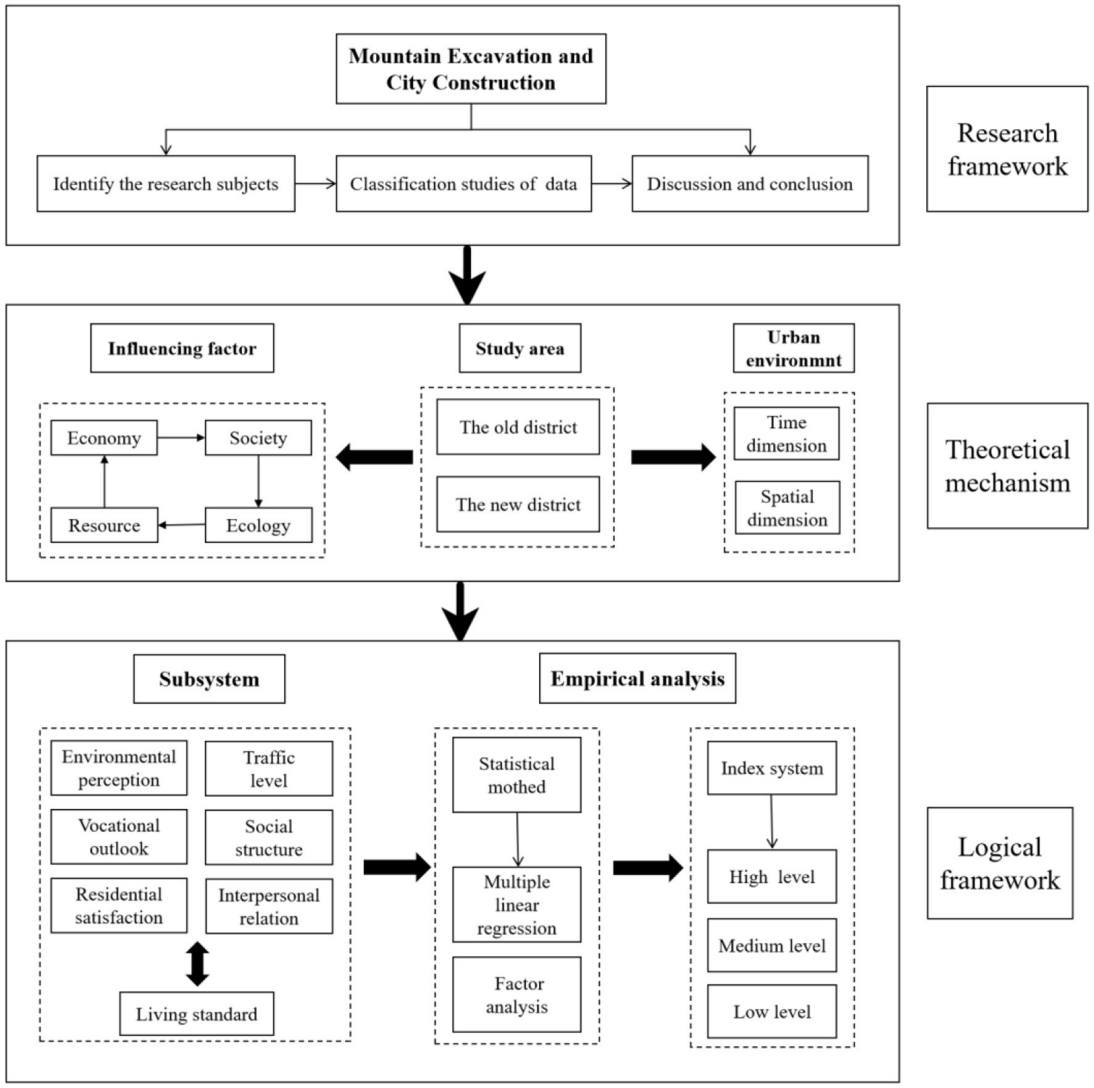
Study architecture diagram.

Over the course of a decade, and with an investment totaling billions, the monumental endeavor known as the Yan'an MECC project has reshaped the landscape by flattening 33 major mountains. This transformative initiative not only symbolizes a significant financial commitment but also signifies a profound dedication to enhancing urban livability. The MECC project presents fresh avenues for advancing research in urban livability. Therefore, this study embarks on a comprehensive analysis aimed at unraveling the multifaceted impact of MECC engineering on urban livability. The study architecture diagram of this study is shown in Figure 1. To achieve this objective, the whole work was carried out regarding the following steps:

- Data collection was executed through a questionnaire. Rigorous reliability and validity tests were conducted on the measurement scale, confirming the questionnaire's high reliability.
- Constructing a new urban livability appraisal system to evaluate the implementation effect of MECC.
- Using the index of living standard in the evaluation system as the dependent variable and introduced other dimensions of the evaluation system for multiple linear regression.

The main contributions of this paper are in two aspects. Firstly, it offers a distinct data source compared to previous research efforts. The MECC program serves as a global exemplar in urban development and introduces fresh perspectives for enhancing urban livability. Our data, acquired through questionnaire surveys, transcend mere statistical records, offering a more comprehensive reflection of residents' genuine sentiments and requirements. Secondly, we have devised a comprehensive evaluation index system based on these survey findings. The study establishes a comprehensive evaluation index system for urban livability, considering factors such as age, educational background, occupation, and overall respondent perspectives. This index system provides a scientific framework to assess the effectiveness of the MECC project and offers valuable insights for regional planning and socioeconomic development.

## 2 Literature review

Urban livability refers to urban environments that offer high quality of life, encompassing factors such as economic development, urban safety, public facilities, transportation conditions, and cultural environment (16, 17). Some researchers find what makes areas livable by analyzing critical factors across diverse districts, while others concentrate on varying elements within a decided zone (18, 19). Presently, the process of urbanization has been accelerating, and the quality of urban life has become an important issue of concern. The construction of urban buildings and infrastructure directly affects the quality of life of urban residents (20).

Engineering construction enhances the quality of housing infrastructure and improves access to essential services such as water, electricity, and transportation. These enhancements lead to greater comfort, convenience, and overall satisfaction among residents with their living conditions. Engineering endeavors also influence residents' environmental perceptions (21). Environmental perception is primarily examined through analyses of both the living environment and human factors (22, 23).

The construction of urban buildings and infrastructure profoundly impacts residents' living standards. Improved transportation systems, healthcare facilities, and educational resources contribute to elevated living standards, fostering wellbeing and satisfaction among residents (24). Surveys have revealed a strong correlation between the quality and utilization efficiency of public facilities such as transportation and sports amenities in urban areas and residents' happiness (25). This correlation stems from the ability of these supportive facilities to meet residents' daily living needs, enhancing convenience and effectively raising living standards. Additionally, higher income levels and reduced living costs can bolster residents' willingness to continue residing in a particular area, thereby positively influencing living standards (26, 27).

Furthermore, the construction of infrastructure also has an impact on livability in terms of ecology environment. Residents' perceptions of green spaces, air and noise pollution, safety and security, and access to public facilities profoundly affect their quality of life and sense of community (28). For instance, with the exacerbation of summer thermal conditions due to global climate change, outdoor amenities play a crucial role in mitigating heat stress for residents (29–31). Consequently, enhanced access to improved infrastructure, amenities, and services can enhance residents' quality of life.

Urban construction should prioritize residents' needs and preferences to foster sustainable, inclusive, and equitable communities that enhance urban livability. Social structure has been increasingly recognized as a crucial factor in shaping urban livability. Public facilities significantly impact social structure by shaping residents' social interactions and opportunities. Thoughtful allocation and design of facilities like sports complexes, commercial centers, and transportation networks can bolster social cohesion, spur economic growth, and enhance urban livability (32–34). Urban engineering projects typically introduce new social resources and infrastructure, such as roads and cultural amenities, whose establishment and layout directly influence social structure (35).

Research indicates that interpersonal relationships play a significant role in urban livability. A sense of belonging and social connectedness is essential for personal wellbeing and quality of life (36). Neighborhood factors such as social cohesion, trust, and perceived safety also influence residents' satisfaction with their living environment (37, 38). Conversely, social deprivation and isolation have adverse effects on mental health and residential satisfaction (39, 40). Therefore, a positive social environment can enhance urban livability, while a negative social environment can detract from it. Policymakers and urban planners should prioritize social connectivity in planning and design to foster resident engagement and satisfaction.

**Figure 2:**
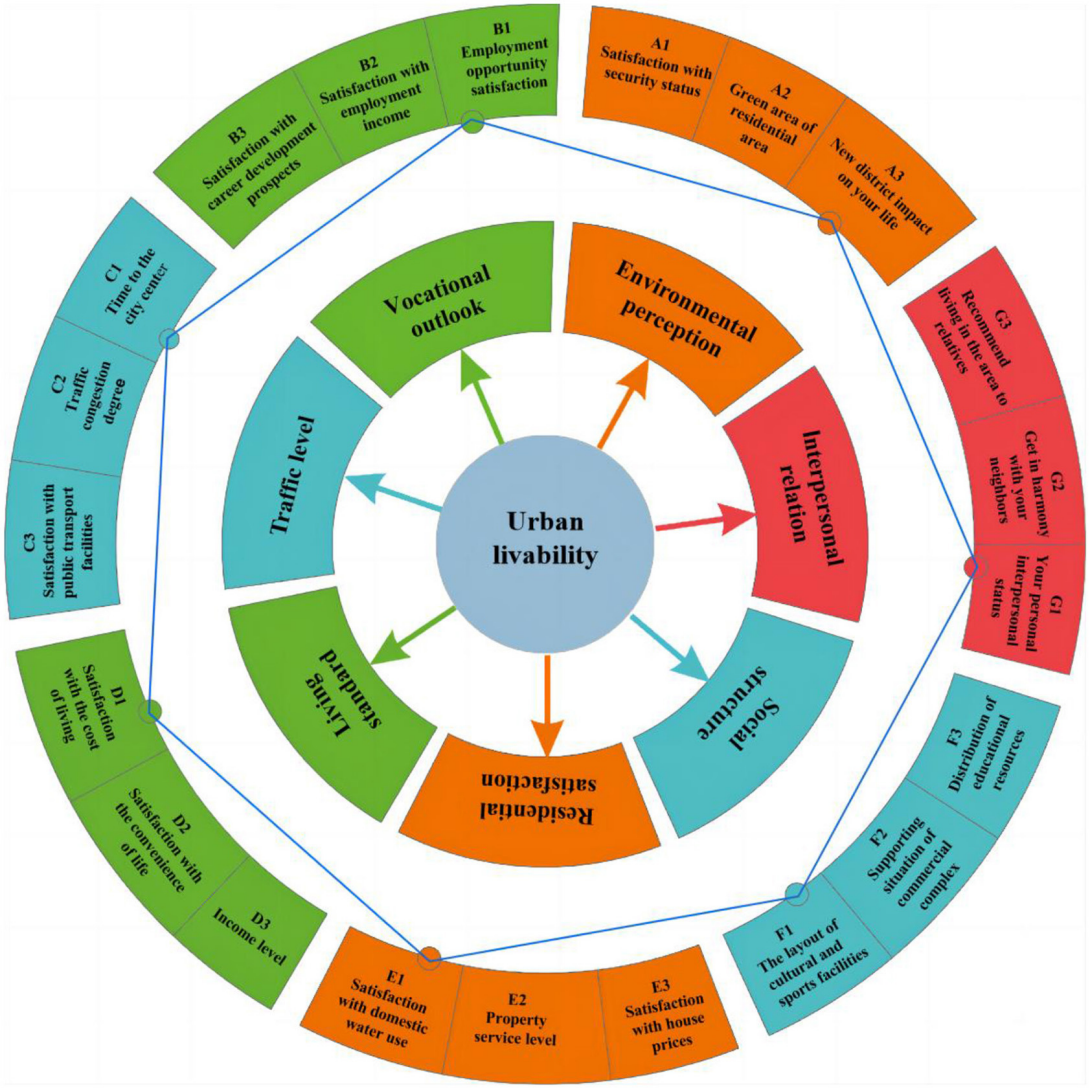
Conceptual framework of urban livability proposed.

In summary, urban livability encompasses various dimensions that collectively contribute to the overall wellbeing and satisfaction of city residents. These dimensions typically include environmental quality, social infrastructure, economic opportunities, transportation, public services, safety, and cultural amenities. However, there are potential limitations to current research on urban livability. Existing studies assessing livability tend to be subjective due to differing perceptions and preferences among individuals and communities. Moreover, existing index systems for evaluating urban livability may not adequately capture the complexity of the urban environment. Therefore, this paper adopts a questionnaire approach to analyze urban livability in the context of MECC program, thereby mitigating the subjectivity of the researcher. These subjective measurements offer valuable insights into human perceptions and emotional connections to the city, facilitating a more comprehensive assessment of livability. The conceptual framework of urban livability proposed in this study is shown in Figure 2.

## 3 Methods and data

### 3.1 Study area

**Figure 3:**
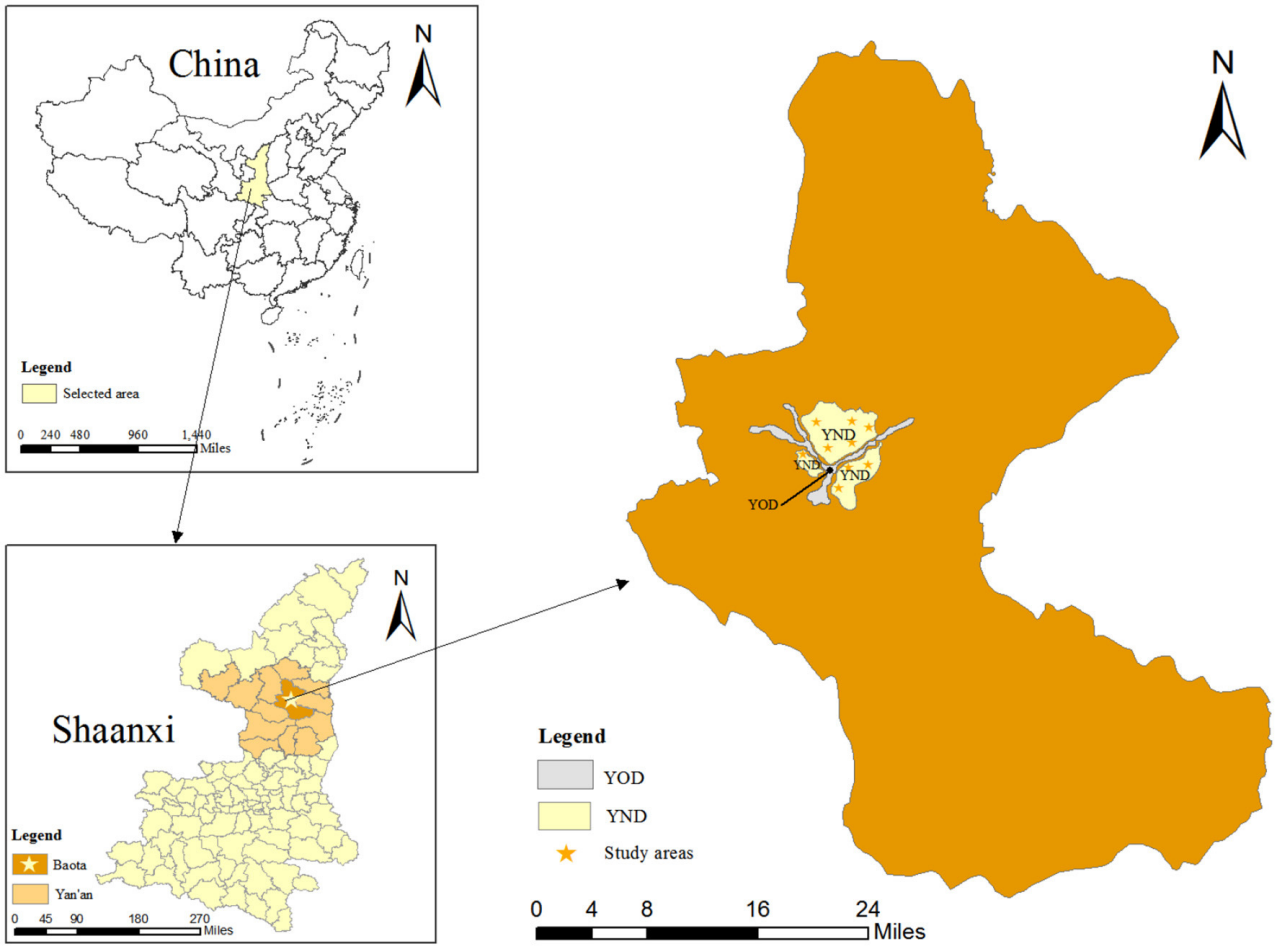
The geographical location of Yan'an.

Yan'an City is a typical squeezed city in China and faces the challenge of limited living space. It situated in the northern region of Shaanxi Province, exhibits an urban morphology characterized by a narrow and elongated pattern along the river valley. The widest zone in the old district (OD) is < 1 km, but the length is more than 20 kilometers. Local residents face the problem of insufficient living space, due to the limitation of geographic space and the continuous growth of population. Thus, the expansion of new urban areas emerges as a pivotal endeavor aimed at enhancing the quality of urban life. The new district (ND) construction plan came under this background. According to this plan, the ND is delineated into three principal zones: north, east, and west. ND primarily serves as a hub for public services, financial and commercial activities, and high-tech industries (The official website of Yan'an New District Management Committee). The geographical layout of ND is depicted in Figure 3.

### 3.2 Data sources

The primary focus of this study is to assess the impact of the MECC. The psychological perceptions of residents regarding the urban environment were integrated into the livability evaluation index system. The study operated on the premise that respondents possessed experiences in both the ND and OD, allowing for a comparative analysis of the data.

The article is based on data collected in 2021 through field interviews with residents in Yan'an. Regarding the questionnaire data obtained, SPSS was utilized to handle individual missing data. The missing data were replaced by the average substitution method of adjacent points to obtain 266 valid samples. As depicted in Table 1, the majority of respondents are young or middle-aged individuals. Additionally, 62.03% of respondents' household registrations are in towns, with over half being married. Furthermore, 81.96% of respondents possess a high school degree or higher education level. In terms of occupation, the primary categories include public institutions (18.05%), enterprises (20.68%), and freelance occupations (24.06%).

**Table 1:** Characteristics of the respondents.

**Variable**	**Class**	**Frequency**	**Proportion**
Age	< 20	28	10.53%
	20−29	90	33.84%
	30−39	89	33.46%
	40−49	35	13.16%
	50−59	17	6.39%
	>60	7	2.63%
Household registration	1 Town	165	62.03%
	2 Rural areas	101	37.97%
Record of formal schooling	1 Primary school below	1	0.38%
	2 Primary school	7	2.63%
	3 Junior high school	40	15.04%
	4. Senior high school	52	19.55%
	5 Junior college or above	166	62.41%
Health condition	1 Extremely poor	0	0.00%
	2 Poor	1	0.38%
	3 General	45	16.92%
	4 Better	100	37.59%
	5 Extremely good	120	45.11%
Marital status	1 Unmarried	115	43.23%
	2 Married	143	53.76%
	3 Divorce	6	2.26%
	4 Other	2	0.75%
Personal occupation	1 Governmental agencies	25	9.40%
	2 Public institutions	48	18.05%
	3 The enterprise	55	20.68%
	4 Freelance	64	24.06%
	5 Retired	4	1.50%
	6 Students	43	16.17%
	7 Other	27	10.15%

### 3.3 Design of the questionnaire

#### 3.3.1 Score standards

To entirely reveal residents' perspectives of MECC, the questionnaire for this research was surveyed according to the standards listed in Table 2. The reply to each issue ranged from 1 (most negative) to 5 (most positive).

**Table 2:** Score indexes of each question.

**Value**	**Meaning**	**Explanation**
1	Extremely low	Be negative about development completely
2	Low	Be negative about development partly
3	Neither low nor high	Opinions or descriptions are neutral
4	High	Be positive about development partly
5	Extremely high	Be positive about development completely

#### 3.3.2 Evaluation system

The concept of urban livability can be explained and analyzed in multiple dimensions, including environmental perception, vocational outlook, traffic level, living standard, residential satisfaction, social structure, interpersonal relation (4, 41). However, in the context of significant engineering projects and their impact on urban livability, there is a need for further investigation. Specifically, it is essential to explore how these projects influence the overall livability of cities. The study set up a set of questionnaires to assess urban livability and compared the effects of MECC on the lives of residents. The designed evaluation system consists of 21 measurement items. The questionnaire design is shown in Table 3. By administering this questionnaire to a representative sample of residents, the study aims to provide valuable insights into the impact of the MECC on urban livability. The findings will shed light on which dimensions are positively or negatively affected and help identify areas that require attention and improvement.

**Table 3:** Specific questions of questionnaire.

**Dimension**	**Questions**	**Scores**
Environmental perception	A1 Satisfaction with security status	()
	A2 Green area of residential area	()
	A3 New district impact on your life	()
Vocational outlook	B1 Employment opportunity satisfaction	()
	B2 Satisfaction with employment income	()
	B3 Satisfaction with career development prospects	()
Traffic level	C1 Time to the city center	()
	C2 Traffic congestion degree	()
	C3Satisfaction with public transport facilities	()
Living standard	D1 Satisfaction with the cost of living	()
	D2 Satisfaction with the convenience of life	()
	D3 Income level	()
Residential satisfaction	E1 Satisfaction with domestic water use	()
	E2 Property service level	()
	E3 Satisfaction with house prices	()
Social structure	F1 The layout of cultural and sports facilities	()
	F2 Supporting situation of commercial complex	()
	F3 Distribution of educational resources	()
Interpersonal relation	G1 Your personal interpersonal status	()
	G2 Get in harmony with your neighbors	()
	G3 Recommend living in the district to relatives	()

Evaluating vocational outlook involves several dimensions such as career development prospects, income satisfaction, and employment opportunity satisfaction (42–44). Aiming to comprehensively evaluate the factors that contribute to a suitable living environment, it is necessary to measure different factors such as public transportation facility satisfaction, traffic congestion levels, and travel time to the city center (45–48). The social structure of urban areas reflects the availability of resources and services such as education, medical care, culture, and entertainment.

Residential conditions are also an important factor in determining living satisfaction. Factors like water supply reliability, management services, and housing cost significantly impact housing satisfaction (49, 50). Living standards can be measured by various indicators such as cost, income, and travel convenience (21, 51, 52). Residents' environmental perception is mainly measured based on factors like public security situation, green area, and the impact of engineering construction on human life (53, 54). The evaluation of interpersonal relationships is mainly based on factors like relationship satisfaction and harmony in getting along with others. Several studies have confirmed that lack of belonging significantly reduces residential satisfaction (55).

### 3.4 Methods

#### 3.4.1 Factor analysis

Factor analysis is the method of statistical to extract generic factors from variable data. Reduce the number of variables by grouping variables of the same essence into a factor, finding hidden representative factors among many variables. As a key branch of the dimension reduction, principal component analysis (PCA) is used to find out the basic elements of multivariate variables. In this study, PCA is used for data processing. The basic steps of PCA are as follows:

Data standardization processing: The “Z-score normalization” method is calculated by the following formula: Where *x* represents the raw data, *x*″ the mean and θ the standard deviation (Equation 1).


(1) $$\begin{array}{l} X^{\prime }=\frac{x-x^{\prime \prime }}{\theta } \end{array}$$

The correlation coefficient matrix (or covariance matrix) of the independent variable is found according to the standardized matrix. Assuming samples *x* and *y*, the covariance is calculated as follows (Equation 2):


(2) $$\begin{array}{l} \mathrm{cov}\left(x,y\right)=\frac{\sum_{i=1}^{n}\left(xi-\bar{x}\right)\left(yi-ȳ\right)}{\left(n-1\right)} \end{array}$$

Where, *cov* (*x, y*)>0 indicates *x* and positive correlation, *cov* (*x, y*) < 0indicates *x* and *y* negative correlation, and *cov* (*x, y*) = 0 indicates unrelated, namely *x* and *y* are independent of each other.

Eigenvalues and eigenvectors of the correlation coefficient matrix were calculated. The eigenvalues are sorted in descending order to retain the largest feature vector, and the original independent variable data is transformed into a new space constructed from the feature vectors.

#### 3.4.2 Multiple linear regression

Linear regression is a type of mathematical analysis that depends on least squares function (linear regression equation) to compute the connection with independent and dependent variables. This function is the linear combination of some model variables (regression coefficient). In the research of real issues, the alteration of dependent variables is often influenced by respective diverse factors. Two or more influencing factors should be used as independent variables to explain the alteration of dependent variables. When more than one independent variable is linearly related to the dependent variable, let *y* be the dependent variable and be the independent variable, the mathematical formula of the model is (Equation 3):


(3) $$\begin{array}{l} Y=b_{0}+b_{1}x_{1}+b_{2}x_{2}+\ldots b_{k}x_{k}+e \end{array}$$

Where, *b*_0_ is the constant term, is the regression coefficient, *e* is the difference between the actual observed value and the fitted value.

## 4 Results

### 4.1 Comprehensive analysis

The reliability and validity tests were performed on the measurement scale, which showed that Cronbach's Alpha are 0.878 in ND and 0.820 in OD, indicating the high reliability of the questionnaire. Test results show a KMO value of 0.861, P of 0.000 in ND; KMO value of 0.837 and P of 0.000 in OD. The results of the KMO and P indicate the high validity of the data, and the questionnaire is suitable for factor analysis. Using principal component analysis for factor extraction, extracting the primary information of the questionnaire (Tables 3, 4). Cumulative contribution rate of ND and OD are 67.02%, 67.87% respectively. Seven dimensions were named environmental perception, vocational outlook, traffic level, living standard, residential satisfaction, social structure, and interpersonal relation. In order to evaluate residents' intuitive feelings after MECC, urban livability is discussed in terms of urban type and seven specific sub-dimensions.

**Table 4:** Factor analysis results of urban livability in OD.

**The OD**	**Dimension**	**Factor loading coefficient**							**Interpreted variance**
Environmental perception	A1	0.526							10.72%
	A2	0.720							
	A3	0.818							
Vocational outlook	B1		0.712						10.59%
	B2		0.747						
	B3		0.768						
Traffic level	C1			0.737					10.36%
	C2			0.793					
	C3			0.640					
Living standard	D1				0.800				9.76%
	D2				0.761				
	D3				0.724				
Residential satisfaction	E1					0.799			9.64%
	E2					0.805			
	E3					0.716			
Social structure	F1						0.731		8.71%
	F2						0.725		
	F3.						0.784		
Interpersonal relation	G1							0.758	7.91%
	G2							0.850	
	G3							0.592	
Extraction method: principal component analysis.									
Rotation method: Quartimax method.									
The a-rotation has converged after 6 iterations.									

### 4.2 Data description

**Figure 4:**
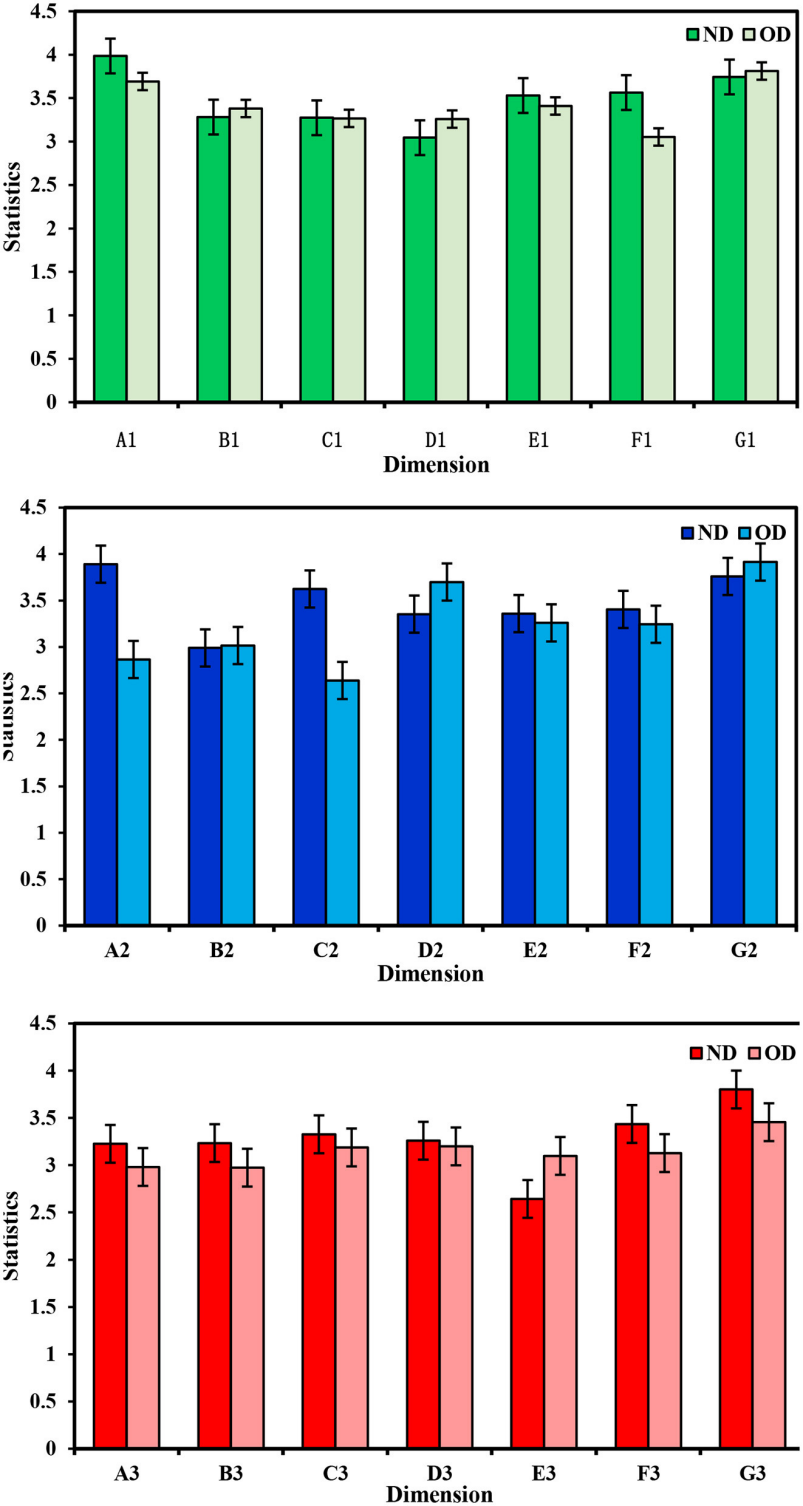
The indicators of questionnaire.

The detailed results of the ND and OD questionnaire data are shown in Tables 4–6 and Figure 4. To explore the specific impact of MECC on Yan 'an, seven subsystems of urban livability were analyzed in this study. Similarly, within the seven dimensions of the livable city evaluation system, five dimensions in the ND scored higher than their counterparts in the OD. Notably, respondents in the ND expressed the highest satisfaction with interpersonal relationships, while their lowest satisfaction pertained to vocational outlook. The average scores for the seven dimensions in the ND ranked as follows, from highest to lowest: interpersonal relationship (3.768), environmental perception (3.701), social structure (3.468), traffic level (3.409), living standard (3.219), residence satisfaction (3.176), and vocational outlook (3.168). In the survey of OD, the dimension with the highest score is interpersonal relationship, and the lowest is traffic level. The average of the seven dimensions of livability in OD are: interpersonal relationship (3.727), living standard (3.386), residential satisfaction (3.253), environmental perception (3.179), social structure (3.142), and vocational outlook (3.123). traffic level (3.031).

**Table 5:** Factor analysis results of urban livability in ND.

**The ND**	**Dimension**	**Factor loading coefficient**							**Interpreted variance**
Environmental perception	A1	0.539							11.15%
	A2	0.763							
	A3	0.774							
Vocational outlook	B1		0.651						10.13%
	B2		0.748						
	B3		0.770						
Traffic level	C1			0.663					9.68%
	C2			0.784					
	C3			0.757					
Living standard	D1				0.697				9.280%
	D2				0.787				
	D3				0.622				
Residential satisfaction	E1					0.720			9.03%
	E2					0.730			
	E3					0.718			
Social structure	F1						0.685		9.02%
	F2						0.555		
	F3						0.710		
Interpersonal relation	G1							0.775	8.74%
	G2							0.836	
	G3							0.614	
Extraction method: principal component analysis.									
Rotation method: Quartimax method.									
The a-rotation has converged after 6 iterations.									

**Table 6:** Statistics for different dimensions of ND and OD.

	**Dimension**	**Statistics of ND**	**Statistics of OD**
Environmental perception	A1 Satisfaction with security status	3.985	3.692
	A2 Green area of residential district	3.891	2.865
	A3 New district impact on your life	3.226	2.981
	Average value	3.701	3.179
Vocational outlook	B1 Employment opportunity satisfaction	3.282	3.381
	B2 Satisfaction with employment income	2.989	3.015
	B3 Satisfaction with vocational outlook	3.234	2.974
	Average value	3.168	3.123
Traffic level	C1 Time to the city center	3.274	3.267
	C2 Traffic congestion degree	3.624	2.639
	C3 Satisfaction with public transport facilities	3.327	3.188
	Average value	3.409	3.031
Living standard	D1 Satisfaction with the cost of living	3.045	3.259
	D2 Satisfaction with the convenience of life	3.353	3.699
	D3 Income level	3.259	3.199
	Average value	3.219	3.386
Residential satisfaction	E1 Satisfaction with domestic water use	3.530	3.410
	E2 Property service level	3.359	3.259
	E3 Satisfaction with house prices	2.643	3.098
	Average value	3.176	3.253
Social structure	F1 The layout of cultural and sports facilities	3.564	3.053
	F2 Supporting situation of commercial complex	3.404	3.244
	F3 Distribution of educational resources	3.436	3.128
	Average value	3.468	3.142
Interpersonal relation	G1 Your personal interpersonal status	3.744	3.812
	G2 Get in harmony with your neighbors	3.759	3.914
	G3 Recommend living in the district to relatives	3.801	3.455
	Average value	3.768	3.727

A comparative analysis of urban livability dimensions between the ND and the OD unveils significant disparities. While the ND showcases notable improvements in residents' environmental perception, vocational outlook, traffic conditions, social structure, and interpersonal relationships compared to the OD, the enhancement in living standards appears less conspicuous. The advancement of infrastructure has substantially augmented residents' travel convenience, thereby fostering a heightened preference for residing in the ND. The disappearance of public space with collective memory and the reduction of storage space and production space also makes the communication between residents in the ND less frequent than in the past, and the unfamiliar interpersonal communication also affects people's experience of the living environment.

### 4.3 Subsystem analysis

**Figure 5:**
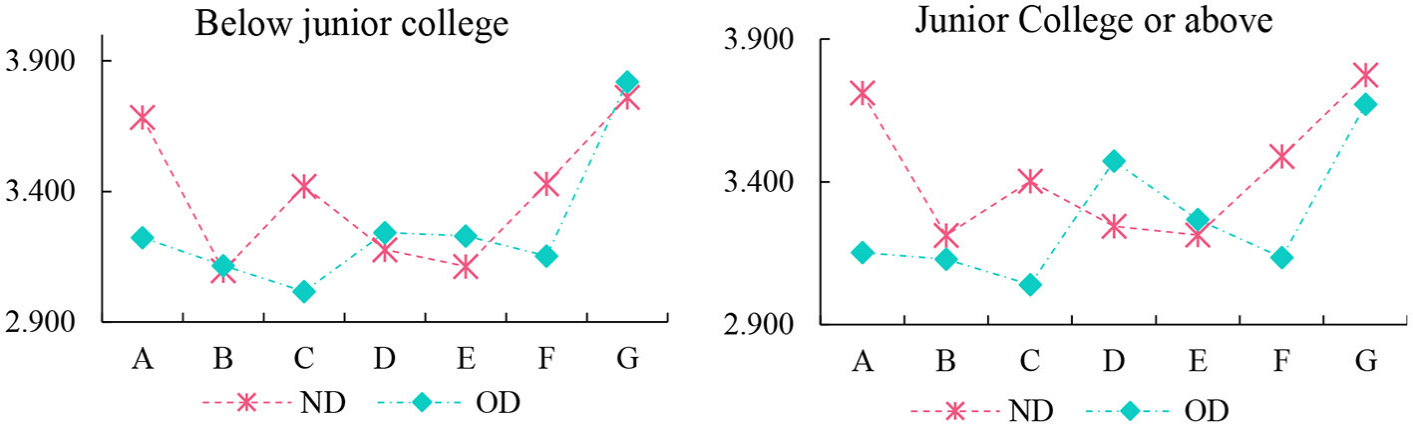
The index of record of formal schooling.

**Figure 6:**
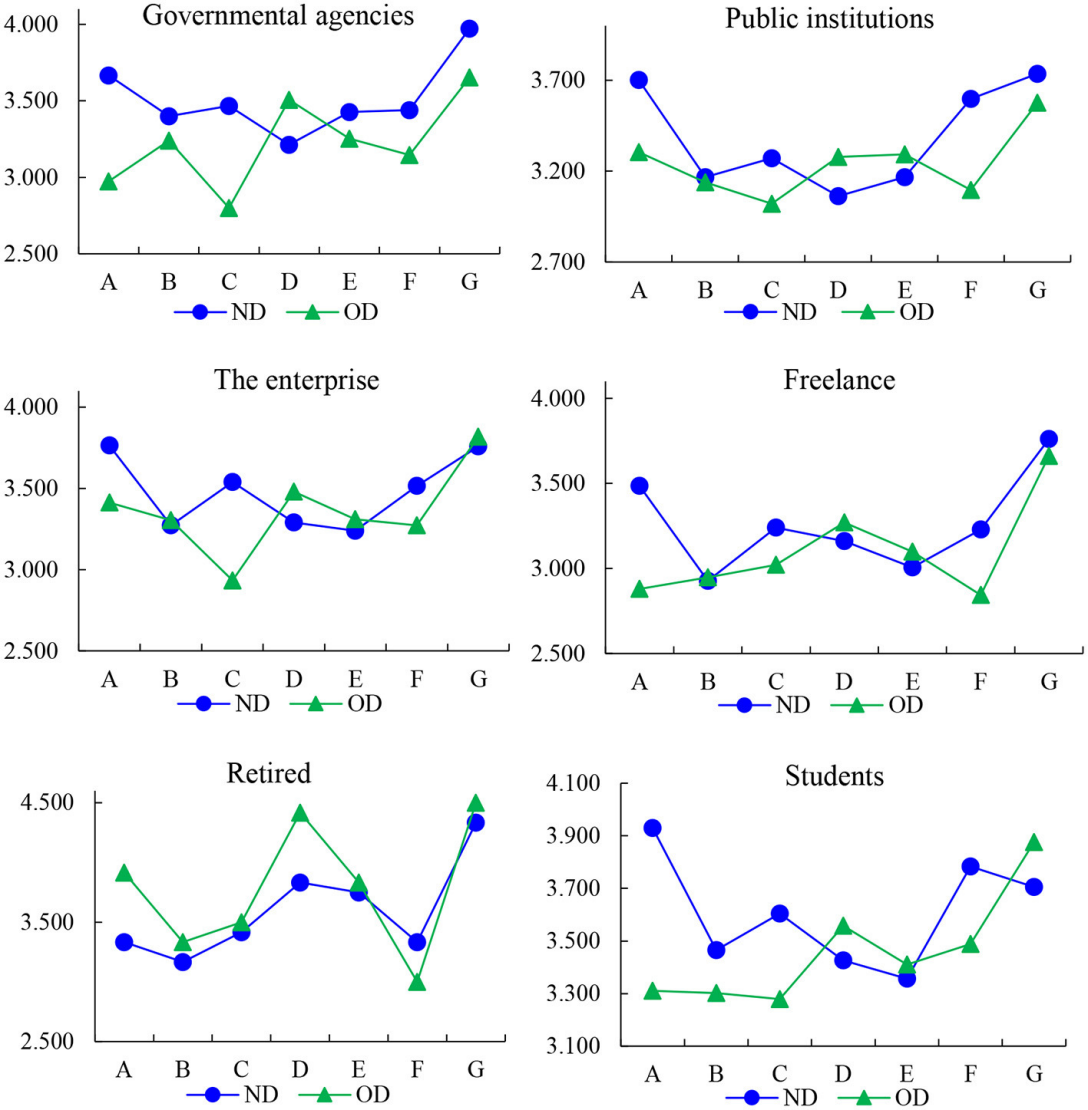
The index of vocation.

**Figure 7:**
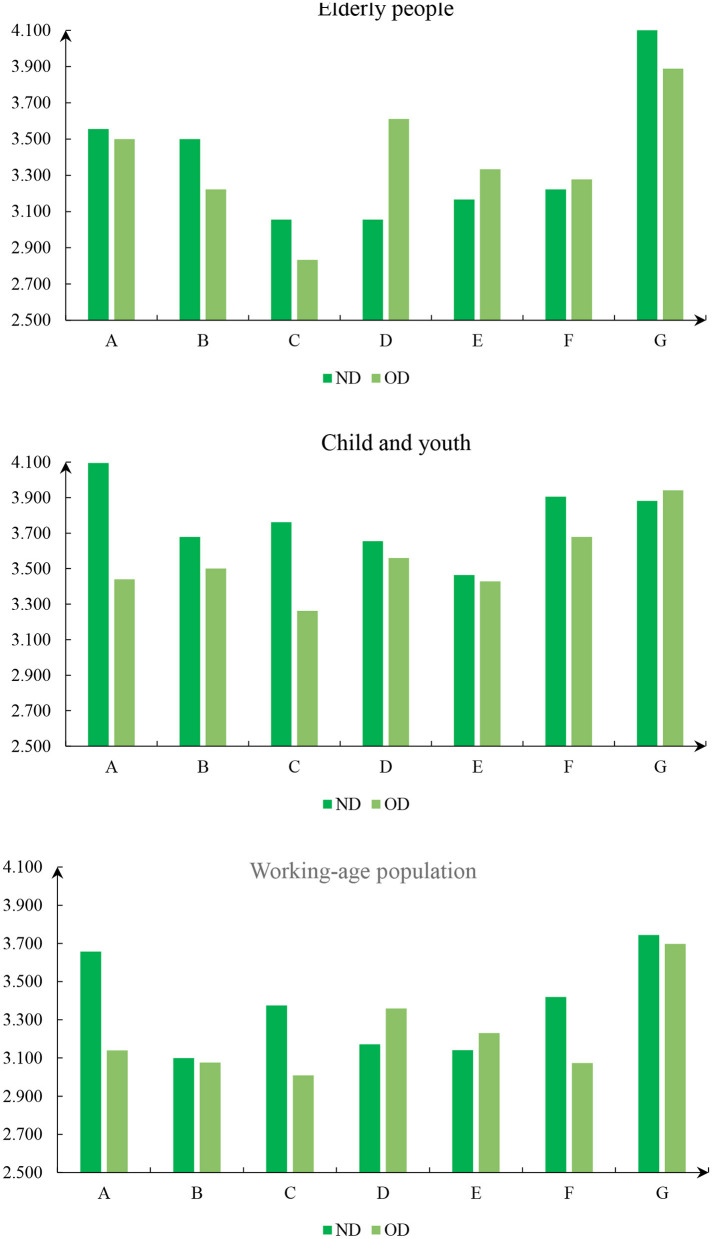
The index of age.

According to the cognitive differences of different types of respondents on the new and old districts, the occupation, age, education and other aspects were analyzed, and the results are shown in Figures 5–7. For the respondents with a college degree or below, MECC effectively improved the environmental perception and traffic level of ND, but the improvement degree was not obvious in other aspects. The college-educated respondents said that the ND was better than OD, except for Residential satisfaction and social structure.

The study found that respondents from government agencies considered that MECC had significantly improved six of the seven dimensions of the index system in ND. However, compared with OD, the living standard score of ND has decreased. Respondents from public institutions and enterprises believe that MECC does not significantly help improve residential satisfaction, vocational outlook, and living standards but improves scores in other dimensions. For freelancers, the living satisfaction and living standard in OD are still higher than those in ND. In the survey, it was found that the retirees only felt that the living standard of ND had been improved to a certain extent. This is mainly because their demand for materials is lower than that for young people. The students thought the living standards and interpersonal relationships of ND were lower than OD. Other respondents, such as security guards, farmers and waiters, believe that the construction of the new district has improved other dimensions other than living standard and Residential satisfaction.

For teenagers below 20 years old, Construction Engineering improved the environmental perception, traffic level, and Living standard in the new district, but the positive impact in other aspects was insignificant. For working-age respondents (20–60 years old), MECC improved the five dimensions of urban livability except for residential satisfaction and social structure. Retirees over the age of 60 believe that the construction has improved the Living standard and Residential satisfaction of the new district but has not shown much help in other fields, which is the opposite of those in the working stage. In summary, the research results highlight variations in the perceived impact of MECC among different age groups. While teenagers and working-age respondents have witnessed significant improvements in more dimensions, retirees have mainly benefited in terms of living standards and residential satisfaction. These findings underscore the importance of considering age-specific needs and preferences when planning and implementing future urban development projects to ensure comprehensive and inclusive enhancements in urban livability.

### 4.4 Affecting factors of living standard

**Figure 8:**
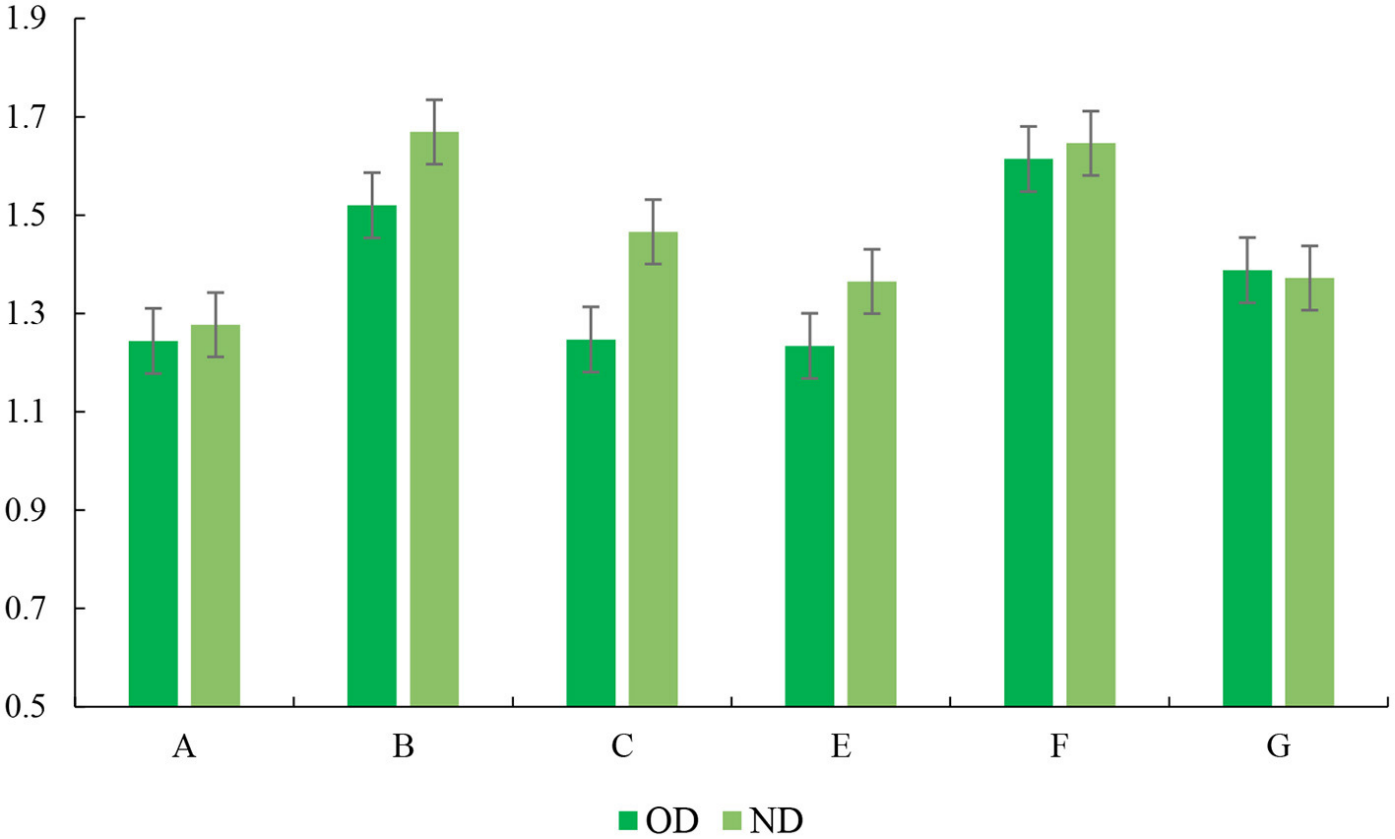
The index of VIF.

**Figure 9:**
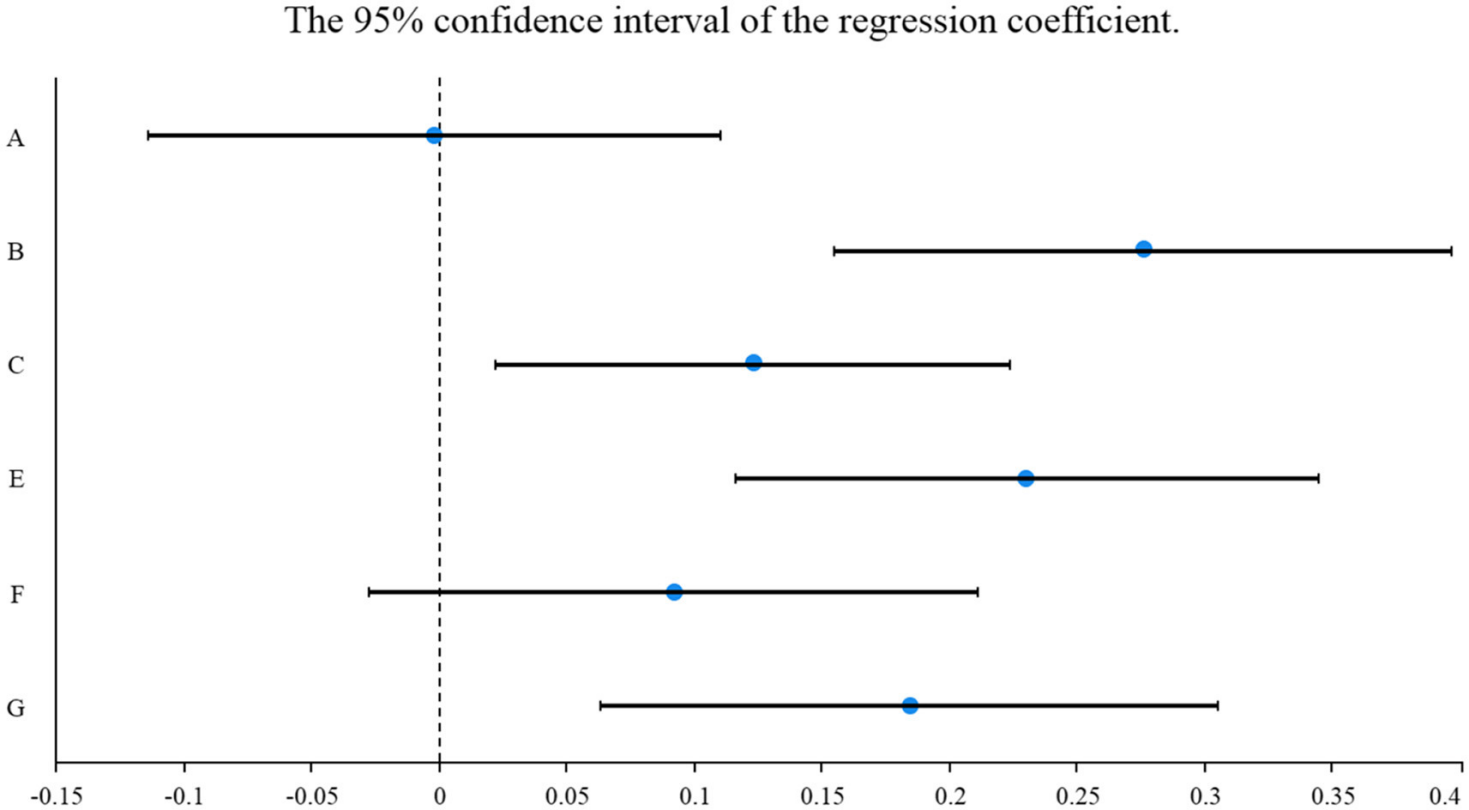
The 95% confidence interval of OD.

**Figure 10:**
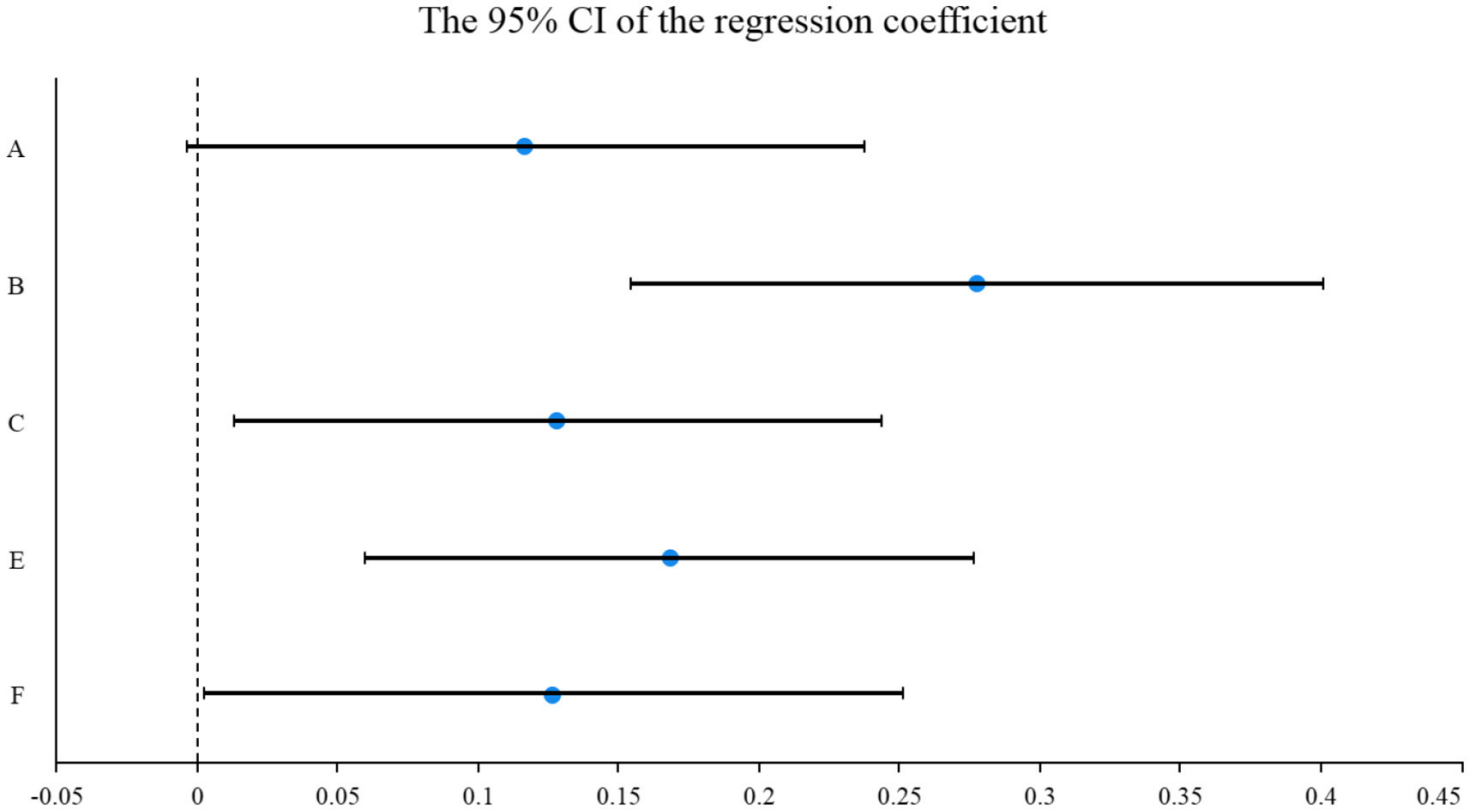
The 95% confidence interval of ND.

As a key element in the urban livability evaluation system, living standards are closely related to residents' daily lives. In order to explore the decisive factors of the issue, the effect of other dimensions on the living standard was analyzed using multiple linear regression models. The dependent variable in the regression analysis was the average of residents' perception of the living standards in ND and OD. And average of the other six dimensions were introduced as the independent variables. The regression results are listed in Tables 7–9, Figures 8–10.

**Table 7:** Summary of the multiple linear regression models.

	**R**	**Adjust to party R**	**Error in the standard estimates**	**Durbin Watson**
The new district	0.607	0.354	0.760	1.958
The old district	0.609	0.357	0.716	1.779
Predictors: (constant), G (interpersonal relationship), C (traffic level)				
A (environmental perception), E (residence satisfaction), F (social structure), B (vocational outlook)				
Dependent variable: D (living standard)				

**Table 8:** Results of the old district linear regression model.

	**Unstandardized coefficients**		**Standardization coefficient**		
	**B**	**Standard error**	**Beta**	**t**	**Conspicuousness**
(Constant)	0.439	0.261		1.678	0.095
A (Environment aware)	−0.002	0.057	−0.002	−0.036	0.971
B (Vocational outlook)	0.276	0.062	0.271	4.463	0.000
C (Traffic level)	0.123	0.051	0.131	2.382	0.018
E (Residential satisfaction)	0.230	0.058	0.215	3.937	0.000
F (Social structure)	0.092	0.061	0.094	1.504	0.134
G (Interpersonal relation)	0.184	0.062	0.173	2.987	0.003
Dependent variable: D (living standard)					

**Table 9:** Results of the new district linear regression model.

	**Unstandardized coefficients**		**Standardization coefficient**		
	**B**	**Standard error**	**Beta**	**t**	**Conspicuousness**
(Constant)	0.262	0.268		0.977	0.329
A (Environment aware)	0.090	0.061	0.082	1.468	0.143
B (Vocational outlook)	0.258	0.062	0.264	4.135	0.000
C (Traffic level)	0.119	0.058	0.122	2.046	0.042
E (Residential satisfaction)	0.124	0.057	0.126	2.178	0.030
F (Social structure)	0.106	0.063	0.107	1.688	0.093
G (Interpersonal relation)	0.170	0.060	0.163	2.822	0.005
Dependent variable: D (living standard)					

The vocational outlook, residence satisfaction, interpersonal relationship, and transportation level significantly impact the living standard. In OD, the influence factors of the above four dimensions on living standards are 0.276, 0.230, 0.184, and 0.123. Affected factors ranging from high to low are vocational outlook (0.258), residential satisfaction (0.124), traffic level (0.119), and interpersonal relationship (0.170) in ND. However, the impact of environmental perception and the social structure was insignificant on residents' living standards in ND and OD. The contrast between the security conditions in different regions is not extraordinarily obvious, which makes the residents not have a deep understanding of the connection between environmental perception and living standards. Moreover, the majority of the sample consisted of young men working in the region. The residents are more focused on own jobs, which makes them less sensitive to the results of urban construction.

The influence coefficient of the vocational outlook on the residents' living standard is 0.276, which is the primary factor affecting the living standard. The results of its development are closely related to personal autonomy, wealth income, social status and other fields. The influence coefficient of residential satisfaction dimension on living standards (0.230) ranked second. Residential conditions are the most basic demand of residents, which is closely related to residents' daily life. The quality of housing, housing price level and the convenience of living environment also significantly affect the residents' perception of own living standards. The influence coefficient of interpersonal relationship and traffic level on residents' living standard was 0.184 and 0.123, respectively, which also had a positive effect. Harmonious neighborhood relationship can promote residents' sense of community belonging, make body and mind more pleasant, and improve residents' living standards. Urban transportation is closely related to residents' daily travel and plays a key role in regional development.

The research results on ND show that the vocational outlook (0.258) is regarded as the critical factor affecting the living standard of the respondents in ND. The ND attracts investment from all parties with excellent infrastructure, public services, and an increasingly perfect urban economic system. It provides a large number of development platforms and work opportunities for residents to work here and is also an essential factor in attracting people to settle here. The impact of relationships on living standards (0.170) rose to second place. The survey found that more young men sought jobs in ND. They tend to regard interpersonal communication as a critical element of work and are more sensitive to the impact of interpersonal relationships on living standards.

## 5 Discussion

The study's findings offer intriguing insights into the varied perceptions of different occupational, age, and educational regarding the impact of MECC. Public institutions, enterprises, freelancers, retirees, and students displayed mixed opinions, highlighting the varying effectiveness of MECC across different dimensions and occupational. The finding underscores the importance of adopting a comprehensive and inclusive approach to urban development projects, one that takes into account the diverse needs and preferences of different occupational to ensure equitable improvements in urban livability (56, 57). Retirees aged over 60 perceive that the construction in the ND has primarily enhanced living standards and residential satisfaction. However, its impact on other dimensions appears limited, suggesting that the project may not have effectively addressed retirees' specific needs and concerns regarding vocational opportunities, social interactions, and environmental perception (16). Respondents with a college education perceived that the ND outperformed the OD in most dimensions, except for residential satisfaction and social structure. While the MECC demonstrated positive outcomes in enhancing certain dimensions for respondents with a college degree or below, further improvements are necessary to address residential satisfaction and social structure (3, 4, 58).

These findings suggest that authorities prioritize regional construction strategies aimed at economic growth, potentially overlooking certain sustainability factors like long-term traffic planning and environmental protection (59, 60). The relevant problems existing in the urban development of OD provide a lesson for the construction of ND and have become a key element for improvement in MECC engineering. The construction of large-scale projects has the potential to significantly alter the surrounding environment due to factors such as land use changes, resource consumption, and pollution emissions (44, 50, 61). It is essential to carefully assess and mitigate these impacts to ensure sustainable development and the preservation of ecological balance. To foster harmonious district development, optimization of industrial structure, enhancement of the social security system, and bolstering pollution prevention are imperative (3, 62–65).

Regional livability significantly influences residents' psychological perception, closely intertwined with ecological, economic, and social security factors (3, 66, 67). Studies indicate that engineering construction profoundly affects urban living environments (68), enhancing living convenience via modern infrastructure and promoting wellbeing through green infrastructure development, which improves air quality and reduces noise levels. However, the MECC engineering is not particularly obvious for the improvement of living standard and residential satisfaction. In the survey, it was found that a rise in house prices led to an enhancement of the cost of living, thereby reducing the willingness of residents to live in the ND. At the same time, the daily cost can be controlled at a relatively reasonable level for the mature public service system in the OD. It is an important reason the residents are more satisfied with the living cost and convenience in the OD. Existing research shows that while economic development improves residents' quality of life, it also induces problems such as shortage of public resources, high cost of living, and ecological degradation (69).

Natural disasters have increased in densely populated areas, resulting in negative awareness of the living environment (70). Studies have shown that well-planned and executed projects can positively impact residents' perceptions of their surroundings (71, 72). The green coverage rate has been improved by constructing parks and urban green belts in ND. While improving residents' living environment, soil erosion has been effectively contained, and the original geological disasters have been eliminated. The researchers found that satisfaction with the city context is linked with elements concerning regional security. Urban security is also a premise for shaping a livable urban environment (73). Engineering construction plays a vital role in transportation development. By investing in transportation infrastructure, cities can alleviate congestion, and stimulate economic growth (44). These developments in urban engineering contribute to the seamless integration of transportation networks and foster the sustainable development of cities. Convenient transportation can provide high mobility for urban residents and save them some travel time, thus improving urban residents' satisfaction with the urban environment (14, 74).

Urban construction significantly influences career prospects. It creates job opportunities in construction, engineering, transportation, and services sectors (75). Additionally, the development of urban infrastructure fosters the growth of knowledge-intensive industries, attracting professionals in technology, finance, and creative sectors. These advances have enhanced employment options, driven innovation and economic growth, thus enhancing the livable nature of cities. Human resources and commercial capital are the basis of urban sustainable development and the embodiment of the comprehensive competitiveness of cities. Through financial support and policy guidance, this zone aims to improve the business environment and expand investment and entrepreneurship. The more capital has promoted the further optimization of the business environment in the capital market, and brought more development platforms and employment opportunities for the residents in this district.

## 6 Conclusions

Based on the analysis of 266 questionnaires and statistical data, this study developed a novel comprehensive urban livability evaluation system. Firstly, factor analysis was employed to quantitatively assess the livability indicators of both ND and OD, thereby examining the tangible impacts of the MECC project. The findings revealed a significant enhancement in urban livability within the ND, particularly evident in improved housing conditions and related supporting facilities following living space renovations. Subsequently, a multiple linear regression model was utilized to investigate the factors influencing residents' living standards. The analysis identified occupational outlook as a significant determinant, with a coefficient of influence of 0.276, highlighting its pivotal role in shaping residents' living standards.

The findings presented in this paper demonstrate the government's successful achievement of its objectives in enhancing residents' living environments and urban construction standards. Additionally, residents from diverse age groups, occupations, and educational backgrounds exhibited varying responses to the MECC program. However, challenges such as industrial restructuring, land resource depletion, and changes in living environments post-relocation have imposed economic and lifestyle pressures on residents. Hence, while the MECC program represents an initial step toward improving residents' living conditions, sustained efforts are required to further enhance urban livability.

The practical implications of this study are significant. Firstly, local governments should address issues of inadequate livability in the ND by prioritizing infrastructure improvements and increasing investment in urban development. Secondly, fostering innovation platforms and creating more and better employment opportunities within the region are essential strategies for attracting and retaining talent. Finally, encouraging citizens with diverse socio-economic attributes to participate in the urban development process is crucial. Understanding and accommodating the varied needs of urban residents is essential for creating truly livable cities.

While this paper has generated several intriguing conclusions, it is important to acknowledge its limitations. The relatively small sample size of 266 questionnaires may restrict the generalizability of the findings. To address this limitation and enhance the robustness of future research, expanding the sample size could be beneficial.

## Data availability statement

The data analyzed in this study is subject to the following licenses/restrictions: Data may be requested from the corresponding author upon reasonable request. Requests to access these datasets should be directed to zhanglanyue@scujj.edu.cn.

## Author contributions

LL: Formal analysis, Funding acquisition, Methodology, Project administration, Validation, Writing—original draft, Writing— review & editing. LZ: Writing—original draft, Writing—review & editing. YG: Conceptualization, Validation, Visualization, Writing—original draft, Writing—review & editing. KR: Data curation, Formal analysis, Methodology, Software, Visualization, Writing—original draft, Writing—review & editing.
